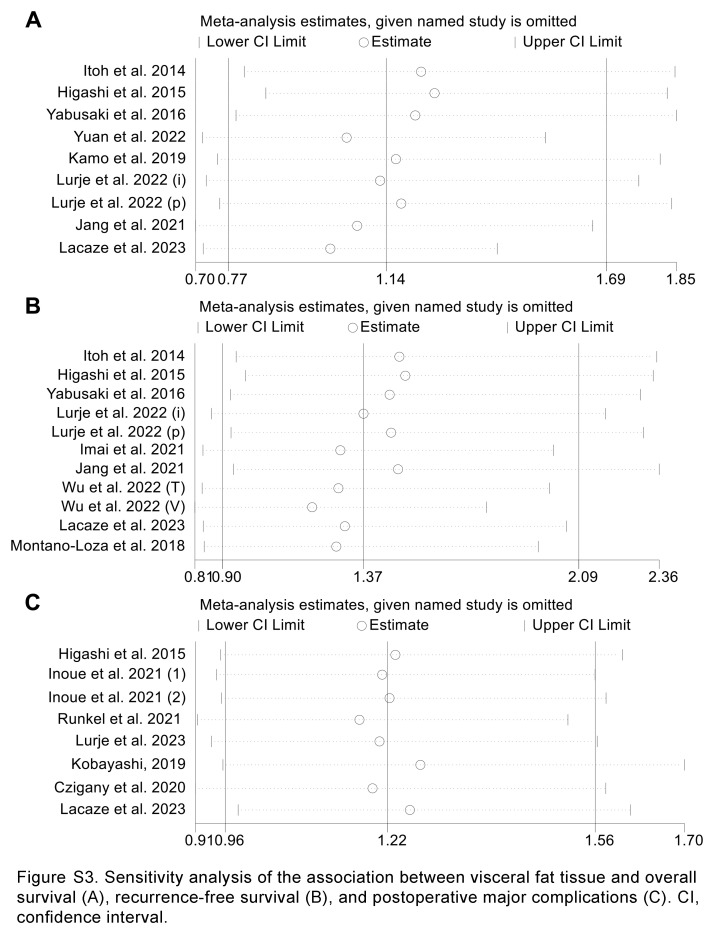# Influence of body fat tissue on outcomes in patients undergoing hepatectomy or liver transplantation: a systematic review and meta-analysis

**DOI:** 10.1097/JS9.0000000000001864

**Published:** 2024-06-26

**Authors:** Lilong Zhang, Zhijia Xia, Zhongyi Li, Jing Zhang, Kunpeng Wang, Weixing Wang

**Affiliations:** a Department of General Surgery, Renmin Hospital of Wuhan University, Wuhan; b Central Laboratory, Renmin Hospital of Wuhan University, Wuhan; c General Surgery Laboratory, Renmin Hospital of Wuhan University, Wuhan, People’s Republic of China; d Department of General, Visceral, and Transplant Surgery, Ludwig Maximilian University of Munich, Munich, Germany; e Division of Basic Biomedical Sciences, The University of South Dakota Sanford School of Medicine, Vermillion, South Dakota, USA

**Keywords:** hepatectomy, intramuscular fat content, liver transplantation, subcutaneous fat tissue, visceral fat tissue, visceral-to-subcutaneous fat tissue ratio

## Abstract

**Objective:**

The purpose of this study is to investigate potential associations between body fat composition and postoperative outcomes in patients with hepatectomy or liver transplantation.

**Methods:**

Three online databases, including Embase, PubMed, and the Cochrane Library, were thoroughly searched for literature describing the relationship between body fat composition and outcomes of patients with liver surgery from the start of each database to 29 October 2023. The Newcastle–Ottawa Scale was used to rate the quality of the studies.

**Results:**

This analysis included a total of 29 articles with a combined patient cohort of 6435 individuals. The results demonstrated that patients with high intramuscular fat content (IMFC) had significantly inferior overall survival (OS) [hazard ratio (HR): 2.07, 95% CI: 1.69–2.53, *P*<0.001] and recurrence-free survival (RFS) (HR: 1.61, 95% CI: 1.20–2.16, *P*=0.002) and a higher risk of major complications (HR: 2.20, 95% CI: 1.59–3.05, *P*<0.001). We also found that the presence of high visceral-to-subcutaneous fat tissue ratio (VSR) in patients with liver surgery was significantly related to poorer OS (HR: 1.70, 95% CI: 1.44–2.00, *P*<0.001) and progression-free survival (PFS) (HR: 1.29, 95% CI: 1.11–1.50, *P*=0.001) and a higher major complication rate (HR: 2.31, 95% CI: 1.17–4.56, *P*=0.016). Besides, the synthesized findings indicated there is no significant correlation between visceral fat tissue and survival outcomes or postoperative complications.

**Conclusion:**

In summary, preoperative IMFC and VSR have the potential to forecast poorer OS and RFS and a higher risk of complications for patients undergoing hepatectomy or liver transplantation.

## Introduction

Highlights

- This study included 29 studies systematically investigating the influence of body fat tissue on outcomes in patients undergoing hepatectomy or liver transplantation.
- The results demonstrated that patients with high intramuscular fat content (IMFC) had significantly inferior overall survival (OS) and recurrence-free survival (RFS) and a higher risk of major complications.
- We also found that the presence of a high visceral-to-subcutaneous fat tissue ratio (VSR) in patients with liver surgery was significantly related to poorer OS and progression-free survival (PFS) and a higher major complication rate.

Traditionally, body mass index (BMI) serves as a simplistic indicator of obesity, with elevated BMI values correlated to increased liver cancer mortality^1^. However, Tateishi *et al*.^2^ found that diminished BMI values predict an unfavorable prognosis in hepatocellular carcinoma (HCC) patients, introducing a paradox known as the ‘BMI paradox’. Analogous paradoxical findings have emerged in other cancer types and cardiovascular diseases^3,4^. The BMI is an indirect measure of body fat; therefore, it may not be able to account for variations in fat distribution, which would explain these inconsistent results. Furthermore, BMI tends to be overestimated in end-stage liver disease patients with substantial ascites and systemic edema^5^.

Computed tomography (CT), dual-energy X-ray absorptiometry, bioimpedance analysis, and magnetic resonance imaging are some of the imaging modalities used in body composition studies to directly quantify skeletal muscle and adipose tissue. Recent reports highlight the capability of axial CT imaging to distinguish between subcutaneous fat tissue and visceral fat tissue, offering a more precise and direct measure of obesity compared to BMI^6^. Visceral adiposity can be quantified through the visceral-to-subcutaneous adipose tissue area ratio (VSR). Besides, intramuscular fat tissue content (IMFC), which is emerging as a novel metric for sarcopenia assessment, provides an effective reflection of skeletal muscle quality^7^. Initially linked to the severity of nonalcoholic steatohepatitis^8^, IMAC correlates with the reduction in both muscle strength and mass due to fatty infiltration of muscle.

Prior research on the influence of baseline body fat composition, such as visceral fat tissue, subcutaneous fat tissue, visceral adiposity, and IMFC, on postoperative outcomes primarily relied on data from individual institutions and focused on specific procedures and pathologies. Moreover, the results appear to contradict each other, indicating incongruous conclusions. Consequently, the transferability and significance of the research are restricted, with insufficient information on the actual impact of body fat composition in the extensive field of liver surgery.

Aggregating data that includes differences in location, organization, and process can assist in addressing this lack of knowledge. Consequently, we performed a comprehensive analysis with the objective of ascertaining the influence of body fat composition on liver surgical results. In conclusion, these findings address the predictive significance of body fat tissue and can assist general surgeons in improving the assessment of preoperative risk and carefully considering patient eligibility.

## Methods

The work has been reported in line with PRISMA (Preferred Reporting Items for Systematic Reviews and Meta-Analyses; Supplemental Digital Content 1, http://links.lww.com/JS9/C880) and AMSTAR (Supplemental Digital Content 2, http://links.lww.com/JS9/C881; assessing the methodological quality of systematic reviews) Guidelines^9,10^.

### Search strategy

Initiated on 29 October 2023, an electronic exploration of diverse bibliographic repositories, including Embase, Cochrane Library, and PubMed, was instigated. This exhaustive exploration employed specific terms such as “hepatectomy” [Mesh], “liver transplantation” [Mesh], “intramuscular fat content”, “visceral fat area”, “visceral fat index”, “subcutaneous fat area”, “visceral adiposity”, “subcutaneous fat index”, and others. This exploration was restricted to studies conducted in the English language involving human subjects. For a more detailed insight into our search strategy, we direct readers to Supplementary Material 1 (Supplemental Digital Content 3, http://links.lww.com/JS9/C882). Additionally, we performed supplementary searches in grey literature using Google Scholar and manually scrutinized the reference lists from qualifying studies. Adhering to the protocols established by the Cochrane collaboration, results from both manual and electronic sources were consolidated using the Covidence software to facilitate effective data management.

### Inclusion and exclusion criteria

We established a predefined set of inclusion and exclusion criteria to guide article selection. The inclusion criteria comprised: (i) The patient underwent hepatectomy or liver transplantation. (ii) Exploration of the prognostic significance of preoperative body fat composition (intramuscular fat content, visceral fat tissue, subcutaneous fat tissue, visceral-to-subcutaneous fat tissue ratio). (iii) The above indicators are divided into two groups: high and low. (iv) At least one of the specified endpoints must be met, such as overall survival (OS), recurrence-free survival (RFS), major complications (Clavien–Dindo grade ≥III), and complications after surgery. RFS was defined as the time from the date of resection to the date the first recurrence was diagnosed or the date of death from any cause. Exclusion criteria encompassed: (i) Exploration of alternative study types, such as animal studies, reviews, case reports, or conference abstracts. (ii) Investigations lacking essential data necessary for computing hazard ratios (HR) or odds ratios (OR) related to the specified endpoints within both the textual content and publicly available records. (iii) In instances where multiple studies included overlapping patient cohorts, preference was given to articles providing comprehensive data and adhering to rigorous methodological standards.

### Data extraction and quality assessment

Throughout the data extraction process, we systematically compiled exhaustive information, encompassing diverse details such as authorship, publication year, study characteristics (region, period, and design), diseases, treatments, demographic features (including sample size, age, and gender distribution), outcomes, method and site assessments, and defined cut-off points. HR and their corresponding 95% confidence intervals (CIs) were predominantly derived from multivariate analyses. In instances where these statistical values were unavailable, recourse to univariate analysis or utilization of the Engauge Digitizer software facilitated data extraction from survival analysis graphs.

To assess the quality of observational studies, we applied the Newcastle–Ottawa Scale (NOS) score. Studies achieving a score of six points or higher were classified as high quality. It is noteworthy that each stage of this procedure, spanning literature retrieval, screening, data retrieval, and quality evaluation, underwent thorough and independent execution by a triad of investigators. Any discrepancies or contentions were resolved through referral to the senior author for adjudication.

**Figure 1:**
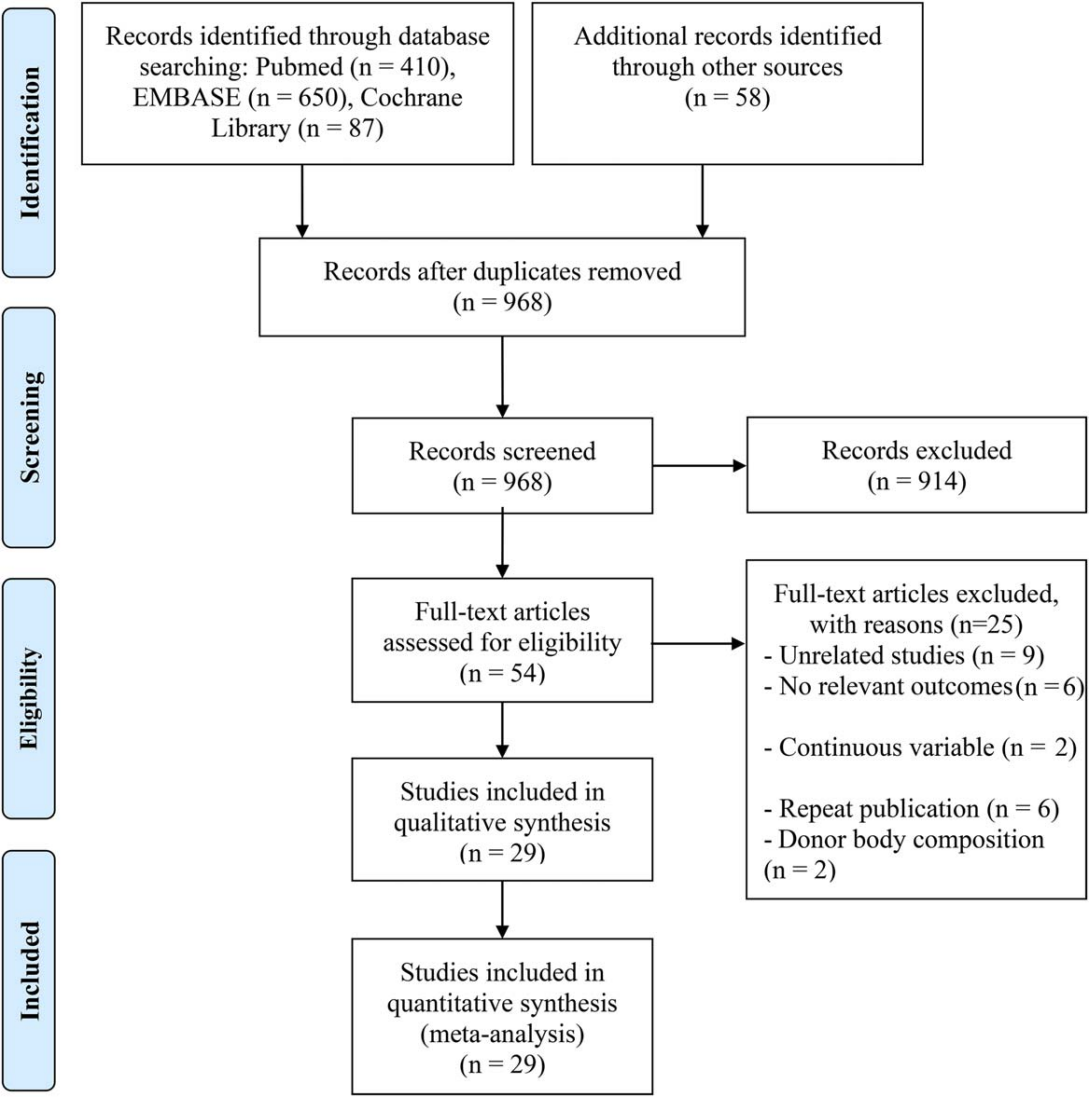
The flow diagram for identifying eligible studies.

### Statistical methods

In this investigation, we employed Stata 15.0 software for statistical analyses. Forest plots were generated for data representation. To assess heterogeneity, Cochran’s *Q* test and *I* ^2^ statistics were utilized, considering either “*P*-value <0.1” or “*I* ^2^ value >50%” indicative of significant heterogeneity. In instances of substantial heterogeneity, a random-effects model was adopted, employing the DerSimonian–Laird method. Conversely, in the absence of notable heterogeneity, a fixed-effect model was applied using the Inverse Variance method. Potential publication bias was evaluated through Egger’s test^11^ and Begg’s test^12^. For results with publication bias, we performed a trim and filling method to assess the impact of missing studies on the pooled results. A sensitivity analysis was systematically conducted to assess the robustness of our findings by iteratively excluding each individual study. Additionally, subgroup analyses were performed, considering factors such as the Cox model, treatments, and visceral fat calculations for comprehensive insights.

## Results

### Literature assessment and selective inclusion of studies

As depicted in the PRISMA flowchart, initially, 1205 research papers were identified through database queries and manual exploration. Following the elimination of duplicate entries, 968 unique articles were retained (Fig. 1). A detailed examination of titles and abstracts led to the exclusion of 914 articles that did not satisfy the eligibility criteria. Out of the remaining selection, 54 articles underwent a thorough full-text review, culminating in the inclusion of 29 articles (31 studies) meeting the predefined criteria for analysis^13–41^.

### Study characteristics

Table 1 presents an outline of the principal characteristics of the studies incorporated in this analysis. The research encompassed a cohort of 7521 participants, with a male prevalence of 72.13%. Geographically, Japan hosted 15 studies, Germany six, China four, and Korea two. Canada, France, Poland, and Turkey each hosted one study. Twenty-two studies included patients undergoing hepatectomy and nine studies included patients undergoing liver transplantation. Of these, 16 studies were diagnosed with HCC, five with colorectal liver metastases (CRLM), six with liver disease (LD), and three with intrahepatic cholangiocarcinoma (ICC). The measurement of body fat composition was conducted using CT scans at the 3th lumbar vertebra in all studies. Additionally, the NOS scores for the 31 studies ranged from 6 to 8 (Table 1).

**Table 1:** Main characteristics of the studies included.

References	Study design	Study period	Study region	Diseases	Treatments	Sample size	Age	Gender (male/female)	Outcome	Cut-off	Method and site	NOS
Itoh *et al*.^22^	R	2004–2009	Japan	HCC	Hepatectomy	190	—	146/34	VFA (OS, RFS, AC)	103 cm^2^ for men and 69 cm^2^ for women	CT, L3	7
Jang *et al*.^34^	R	05/2003–12/2011	Korea	HCC	Hepatectomy	160	55.2±11.5^a^	120/40	VFI (OS, RFS)	30.39 cm^2^/m^2^ for men and 44.70 cm^2^/m^2^ for women	CT, L3	7
Higashi *et al*.^23^	R	2008–2012	Japan	HCC	Hepatectomy	215	67.7±9.3^a^	151/54	VFA (OS, RFS, MC); SFI (OS)	VFA, 103 m^2^ for men and 69 cm^2^ for women; SFI, –	CT, L3	7
Runkel *et al*.^36^	R	01/2012–08/2018	Germany	CRLM	Hepatectomy	94	61.4 (34–83)^b^	58/36	VFA, VSR (AC, MC)	VFA, 168 cm^2^ for men and 80 cm^2^ for women; VSR, 0.4	CT, L3	6
Kaibori *et al*.^24^	R	01/2006–02/2010	Japan	HCC	Hepatectomy	141	76^c^	107/34	IMFC (OS, RFS, AC)	−0.34for men and −0.46 for women	CT, L3	7
Shi *et al*.^41^	R	06/2017–04/2022	China	HCC	Hepatectomy	245	56 (50–64)^d^	198/47	IMFC, VSR (RFS)	–0.324 for men and −0.138 for women 0.63 for men and 1.68 for women	CT, L3	7
Hamaguchi *et al*.^25^	R	04/2005–10/2014	Japan	HCC	Hepatectomy	492	68±10^a^	403/89	IMFC (MC)	−0.324 for men and −0.138 for women	CT, L3	8
Lurje *et al*.^40^	R	03/2010–12/2020	Germany	ICC	Hepatectomy	173	65 (23–83)^b^	86/87	VFA (MC)	100 cm^2^	CT, L3	7
Yabusaki *et al*.^26^	R	07/2003–10/2014	Japan	HCC	Hepatectomy	195	66 (22–80)^b^	157/38	VFA (OS, RFS)	103 cm^2^ for men and 69 cm^2^ for women	CT, L3	7
Kobayashi *et al*.^27^	R	01/2005–09/2014	Japan	CRLM	Hepatectomy	122	65 (59–70)^d^	78/46	IMFC, VSR (OS, RFS, MC)	IMFC, −0.358 for men and −0.229 for women; VSR, 1.325 for men and 0.710 for women	CT, L3	7
Lacaze *et al*.^39^	R	01/2004–11/30	France	ICC	Hepatectomy	91	55^c^	—	VFI, SFI (OS, RFS); VFI (MC)	VFI, 50 cm^2^/m^2^; SFI, 50 cm^2^/m^2^	CT, L3	7
Hamaguchi *et al*.^28^	R	04/2005–03/2016	Japan	HCC	Hepatectomy	606	68 (61–75)^d^	484/122	IMFC, VSR (OS, RFS); VSR (MC)	IMFC, −0.358 for men and −0.229 for women; VSR, 1.325 for men and 0.710 for women	CT, L3	8
Kobayashi *et al*.^29^	R	04/2005–03/2015	Japan	HCC	Hepatectomy	465	67.6±9.6^a^	367/98	VFA (MC)	100 cm^2^	CT, L3	7
Horii *et al*.^30^	R	01/2009–12/2016	Japan	CRLM	Hepatectomy	115	67 (27–85)^d^	79/36	IMFC (OS, RFS, MC)	−0.358 for men and −0.229 for women	CT, L3	7
Lurje *et al*.^37^ (i)	R	05/2010–12/2019	Germany	ICC	Hepatectomy	86	65±11.4	37/49	VFA (OS, RFS)	202.1 cm^2^ for men and 78.0 cm^2^ for women	CT, L3	7
Lurje *et al*.^37^ (p)	R	05/2010–12/2019	Germany	PCC	Hepatectomy	103	66±10.4	71/32	VFA (OS, RFS)	202.1 cm^2^ for men and 78.0 cm^2^ for women	CT, L3	7
Shiozawa *et al*.^31^	R	2008–2016	Japan	CRLM	Hepatectomy	47	62.4±10.6^a^	28/19	IMFC (OS, RFS, MC)	−0.426 for men and −0.362 for women	CT, L3	6
Wu *et al*.^38^ (T)	R	01/2018–10/2020	China	HCC	Hepatectomy	192	50.6 (44.0–56.8)^d^	165/27	VFI (RFS)	37.45 cm^2^/m^2^	CT, L3	7
Wu et al.^38^ (V)	R	01/2018–01/2019	China	HCC	Hepatectomy	46	55.4 (45.0–65.3)^d^	42/4	VFI (RFS)	56.82 cm^2^/m^2^	CT, L3	7
Imai *et al*.^32^	R	05/2006–12/2019	Japan	HCC	Hepatectomy	72	72.6±9.1^a^	49/23	VFI (RFS)	100 cm^2^/m^2^	CT, L3	6
Inoue *et al*.^33^	R	01/2010–12/2018	Japan	CRLM	Hepatectomy	314	—	175/139	VFA (MC)	100 cm^2^	CT, L3	7
Okubo *et al*.^35^	R	01/2011–12/2017	Japan	HCC	Hepatectomy	181	67 (34–87)^b^	123/58	VSR (OS, RFS, AC, MC)	1.33 for men and 0.93 for women	CT, L3	8
Hamaguchi *et al*.^13^	R	01/2008–04/2015	Japan	LD	LT	250	54 (43–62)^d^	122/128	IMFC, VSR (OS)	IMFC, −0.358 for men and −0.229 for women; VSR, 1.325 for men and 0.710 for women	CT, L3	8
Montano-Loza *et al*.^14^	R	01/2000–05/2014	Canada	HCC	LT	457	—	457	VAI (RFS)	65 cm^2^/m^2^	CT, L3	7
Grąt *et al*.^15^	R	01/2006–06/2017	Poland	HCC	LT	77	56 (52–61)^d^	59/18	SFI (RFS)	75.5 cm^2^/m^2^	CT, L3	7
Kamo *et al*.^16^	R	01/2008–06/2016	Japan	LD	LT	277	54 (18–69)^b^	134/143	VFA (OS)	100 m^2^	CT, L3	8
Czigany *et al*.^17^	R	05/2010–12/2017	Germany	LD	LT	225	55±16^a^	117/108	VFA (MC)	100 m^2^	CT, L3	6
Yuan *et al*.^19^	R	04/2015–09/2020	China	LD	LT	175	49 (42–54)^d^	142/33	VFA (OS)	85.2 cm^2^	CT, L3	7
Ari *et al*.^20^	R	01/2015–02/2020	Turkey	LD	LT	65	55 (45–63)^d^	48/17	IMFC, VSR (OS)	IMFC, −0.52 for men and −0.48 for women; VSR, 0.645 for men and 0.24 for women	CT, L3	6
Sim *et al*.^21^	R	01/2008–01/2018	Korea	HCC	LT	1386	—	660/726	VSR (OS, RFS)	0.73 for men and 0.31 for women	CT, L3	7
Meister *et al*.^18^	R	05/2010–12/2017	Germany	LD	LT	264	55 (48–61)^d^	175/89	IMFC (MC)	−0.35 for men and −0.32 for women	CT, L3	7

^a^ Mean±standard deviation.

^b^ Median with range.

^c^ Age ≥69 years.

^d^ Median with interquartile range.

AC, any complications; CRLM, colorectal liver metastases; CT, computed tomography; HCC, hepatocellular carcinoma; ICC, intrahepatic cholangiocarcinoma; IMFC, intramuscular fat content; L3, 3th lumbar vertebra; LD, liver disease; LT, liver transplantation; MC, major complications; OS, overall survival; PCC, perihilar cholangiocarcinoma; R, retrospective study; RFS, relapse-free survival; SFI, subcutaneous fat index; VFA, visceral fat area; VFI, visceral fat index; VSR, visceral-to-subcutaneous fat tissue ratio.

### Correlation of IMFC with OS

**Figure 2:**
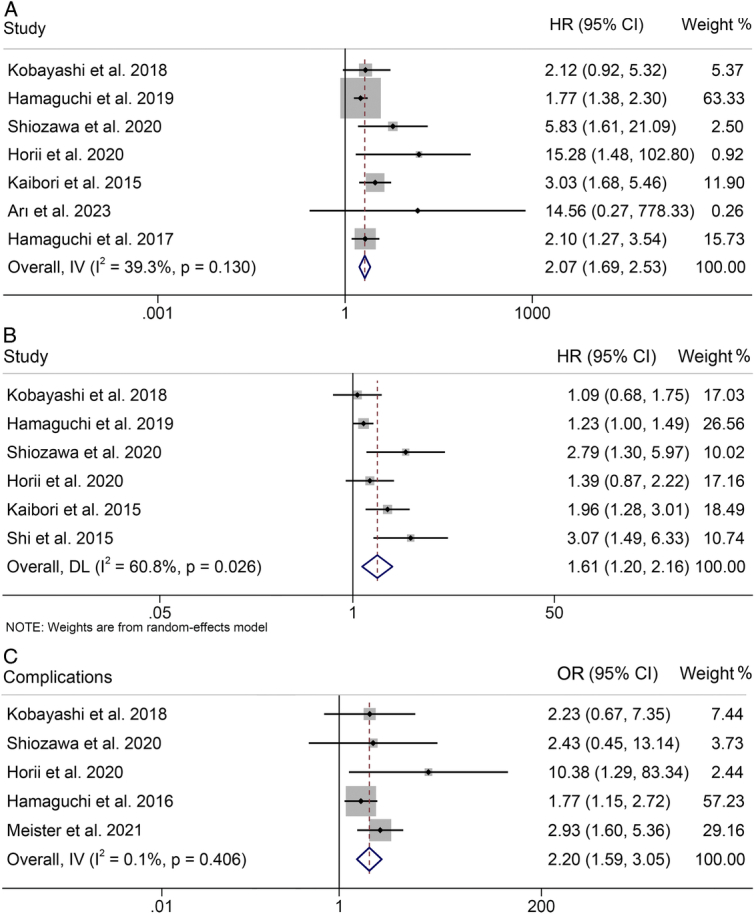
Forest plots of the relationship between intramuscular fat content and overall survival (A), recurrence-free survival (B), and postoperative major complications (C). CI, confidence interval; HR, hazard ratio; OR, odds ratio.

In aggregate, seven studies (involving 1346 patients) probed the influence of baseline IMFC on the OS of patients undergoing hepatectomy or liver transplantation. Cochran’s *Q* test and *I* ^2^ statistics (*I* ^2^=39.3%, *P*=0.130) indicated lower heterogeneity, which led us to apply a fixed-effects model. We observed that individuals with high IMFC manifested a significantly compromised OS (HR: 2.07, 95% CI: 1.69–2.53, *P*<0.001; Fig. 2A) compared to those with low IMFC.

Subgroup analysis, based on treatments and disease types, was conducted. Patients who underwent hepatectomy (HR: 2.69, 95% CI: 1.63–4.46, *P*<0.001) or liver transplantation (HR: 2.17, 95% CI: 1.30–3.60, *P*=0.003) consistently demonstrated that a high IMFC was associated with a shorter OS (Table 2). Besides, we found that high IMFC was associated with worse OS in patients with either HCC, CRLM, or LD (Table 2).

**Table 2:** Subgroup analysis of the association between intramuscular fat content, visceral-to-subcutaneous fat tissue ratio, and the outcomes after hepatectomy or liver transplantation.

		Test of association			Test of heterogeneity	
Variable	Included studies	HR (95% CI)	*P*	Modal	*I* ^2^	*P*
Intramuscular fat content (OS)						
Treatments						
Hepatectomy	5	2.69 (1.63–4.46)	***P*** **<0.001**	R	55.3%	*P*=0.062
Liver transplantation	2	2.17 (1.30–3.60)	***P*** **=0.003**	R	0	*P*=0.345
Cox model						
Multivariate analysis	7	2.07 (1.69–2.53)	***P*** **<0.001**	F	39.3%	*P*=0.130
Disease type						
HCC	2	2.16 (1.30–3.60)	***P*** **=0.003**	R	62.8%	*P*=0.101
CRLM	3	4.29 (1.51–12.14)	***P*** **=0.006**	R	46.2%	*P*=0.156
LD	2	2.17 (1.30–3.60)	***P*** **=0.003**	R	0	*P*=0.345
Intramuscular fat content (RFS)						
Treatments						
Hepatectomy	6	1.61 (1.20–2.16)	***P*** **=0.001**	R	60.8%	*P*=0.026
Cox model						
Multivariate analysis	4	1.94 (1.23–3.08)	***P*** **=0.005**	R	73.9%	*P*=0.009
Univariate analysis	2	1.23 (0.88–1.72)	*P*=0.219	R	0	*P*=0.474
Disease type						
HCC	3	1.78 (1.09–2.93)	***P*** **=0.023**	R	49.5%	*P*=0.121
CRLM	3	1.40 (1.04–1.91)	***P*** **=0.029**	R	76.5%	*P*=0.014
Visceral-to-subcutaneous fat tissue ratio (OS)						
Treatments						
Hepatectomy	3	1.74 (1.39–2.16)	***P*** **<0.001**	F	32.1%	*P*=0.229
Liver transplantation	3	1.66 (1.30–2.10)	***P*** **<0.001**	F	38.2%	*P*=0.198
Cox model						
Multivariate analysis	5	1.67 (1.42–1.98)	***P*** **<0.001**	F	28.4%	*P*=0.232
Univariate analysis	1	2.30 (1.10–4.83)	***P*** **=0.028**	F	–	–
Disease type						
HCC	3	1.63 (1.31–2.04)	***P*** **<0.001**	F	27.4%	*P*=0.252
CRLM	1	2.30 (1.10–4.83)	***P*** **=0.028**	F	–	–
LD	2	2.34 (1.41–3.88)	***P*** **=0.001**	F	0	*P*=0.330
Visceral-to-subcutaneous fat tissue ratio (RFS)						
Treatments						
Hepatectomy	3	1.26 (1.06–1.50)	***P*** **=0.010**	F	0	*P*=0.573
Liver transplantation	2	1.40 (1.04–1.88)	***P*** **=0.026**	F	–	–
Cox model						
Multivariate analysis	3	1.37 (1.16–1.63)	***P*** **<0.001**	F	0	*P*=0.921
Univariate analysis	2	1.05 (0.76–1.44)	*P*=0.790	F	0	*P*=0.818
Disease type						
HCC	4	1.32 (1.12–1.54)	***P*** **=0.001**	F	0	*P*=0.608
CRLM	1	1.09 (0.67–1.77)	*P*=0.727	–	–	–

Bold values indicate statistically significant (P<0.05).

CL, confidence interval; CRLM, colorectal liver metastases; F, fixed-effect model; HCC, hepatocellular carcinoma; HR, hazard ratio; LD, liver disease; OS, overall survival; R, random-effect model; RFS, recurrence-free survival.

### Preoperative IMFC and RFS

In total, six studies, comprising 1276 patients, explored the association between IMFC and RFS in patients with surgical treatment. Acknowledging substantial heterogeneity across the studies (*I* ^2^=60.8%, *P*=0.026), we adopted a random-effects model. The aggregate analysis demonstrated a significantly reduced RFS among patients with high IMFC (HR: 1.61, 95% CI: 1.20–2.16, *P*=0.002; Fig. 2B).

We performed subgroup analyses according to the type of Cox regression analysis and disease types. The superiority of multivariable analysis over univariable analysis lies in its capacity to concurrently assess and control for multiple factors. This enables a more comprehensive and precise analysis, mitigating the impact of confounding variables and enhancing the overall accuracy and robustness of the model. As depicted in Table 2, the multivariable analysis further validated a pronounced reduction in RFS among patients exhibiting elevated IMFC levels (HR: 1.94, 95% CI: 1.23–3.08, *P*=0.005). The above findings also hold true in HCC patients and CRLM patients (Table 2).

### Baseline IMFC and postoperative major complications

The analysis, illustrated in Figure 2C, evaluates the impact of IMFC on the risk of major complications. The *I* ^2^ metric (*I* ^2^=0.1%, *P*=0.406), which revealed the absence of heterogeneity, led to the use of a fixed-effects model. The findings revealed a consolidated OR of 2.20 (95% CI: 1.59–3.05), drawing from four studies involving 1040 patients. This indicates a statistically significant increase in the risk of major complications among individuals with a high IMFC compared to those with a lower IMFC. In addition, these findings held true in both hepatectomised and transplanted patients or in patients with HCC, CRLM, and LD (Supplementary Table S1, Supplemental Digital Content 4, http://links.lww.com/JS9/C883).

### Correlation of VSR with OS

**Figure 3:**
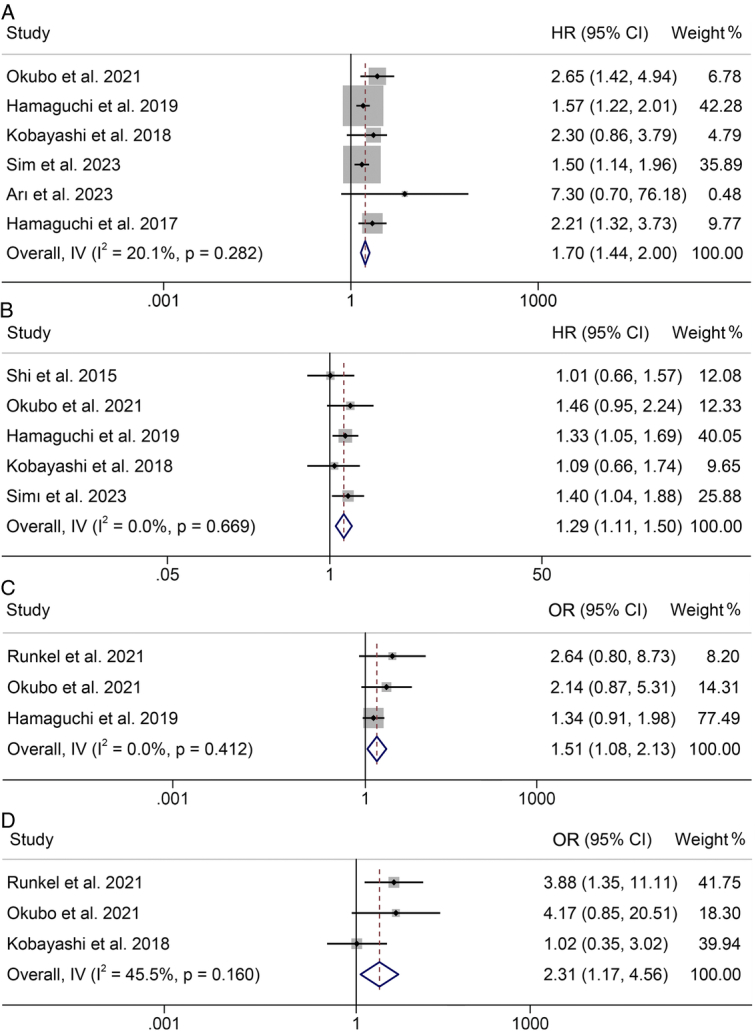
Forest plots of the relationship between visceral-to-subcutaneous fat tissue ratio and overall survival (A), recurrence-free survival (B), postoperative major complications (C), and postoperative complications (D). CI, confidence interval; HR, hazard ratio; OR, odds ratio.

Collectively, six studies involving 2610 patients examined the impact of baseline VSR on the OS of individuals undergoing hepatectomy or liver transplantation. The use of a fixed-effects model was warranted due to lower heterogeneity, as indicated by Cochran’s *Q* test and *I* ^2^ statistics (*I* ^2^=20.1%, *P*=0.282). Our findings revealed a significantly lower OS among individuals with elevated VSR (HR: 1.70, 95% CI: 1.44–2.00, *P*<0.001, as shown in Fig. 3A), in contrast to those with low VSR.

We performed subgroup analysis based on treatments, Cox regression analysis, and disease types (Table 2). The results demonstrated that for individuals undergoing hepatectomy (HR: 1.74, 95% CI: 1.39–2.16, *P*<0.001) and liver transplantation (HR: 1.66, 95% CI: 1.30–2.10, *P*<0.001), the high VSR was associated with markedly diminished OS (Table 2). Both univariate and multivariate analyses consistently indicated that high VSR was linked to a shorter OS (multivariate, HR: 1.67, 95% CI: 1.42–1.98, *P*<0.001; univariate, HR: 2.30, 95% CI: 1.10–4.83, *P*=0.028), reinforcing the robustness of our research findings. Besides, we found that high VSR was associated with worse OS in patients with either HCC, CRLM, or LD (Table 2).

### Preoperative VSR and RFS

Five studies with 2540 patients investigated the correlation between preoperative VSR and RFS in individuals undergoing surgical treatment. There was no heterogeneity among the studies (*I* ^2^=0, *P*=0.669); a fixed-effects model was employed. The comprehensive analysis revealed a notable reduction in RFS among patients exhibiting elevated VSR levels (HR: 1.29, 95% CI: 1.11–1.50, *P*=0.001; Fig. 3B).

Subgroup analysis was conducted considering surgical treatments, Cox regression analysis, and disease types (Table 2). Findings indicated that among individuals undergoing hepatectomy (HR: 1.26, 95% CI: 1.06–1.50, *P*=0.010) and liver transplantation (HR: 1.40, 95% CI: 1.04–1.88, *P*=0.026), elevated VSR was significantly linked to reduced OS (Table 2). Besides, multivariate analysis revealed that high VSR was significantly associated with shorter RFS (HR: 1.37, 95% CI: 1.16–1.63, *P*<0.001).

### Baseline VSR and postoperative complications

The examination depicted in Figure 3C, D assesses the influence of VSR on the occurrence of complications and major complications postoperatively. Our results revealed that patients with high VSR had a significantly higher risk of complications (HR: 1.51, 95% CI: 1.08–2.13, *P*=0.017; Fig. 3C) and major complications (HR: 2.31, 95% CI: 1.17–4.56, *P*=0.016; Fig. 3D) compared to those with low VSR. Subgroup analyses are detailed in Supplementary Table S1 (Supplemental Digital Content 4, http://links.lww.com/JS9/C883).

### Correlation of visceral fat tissue with OS

**Figure 4:**
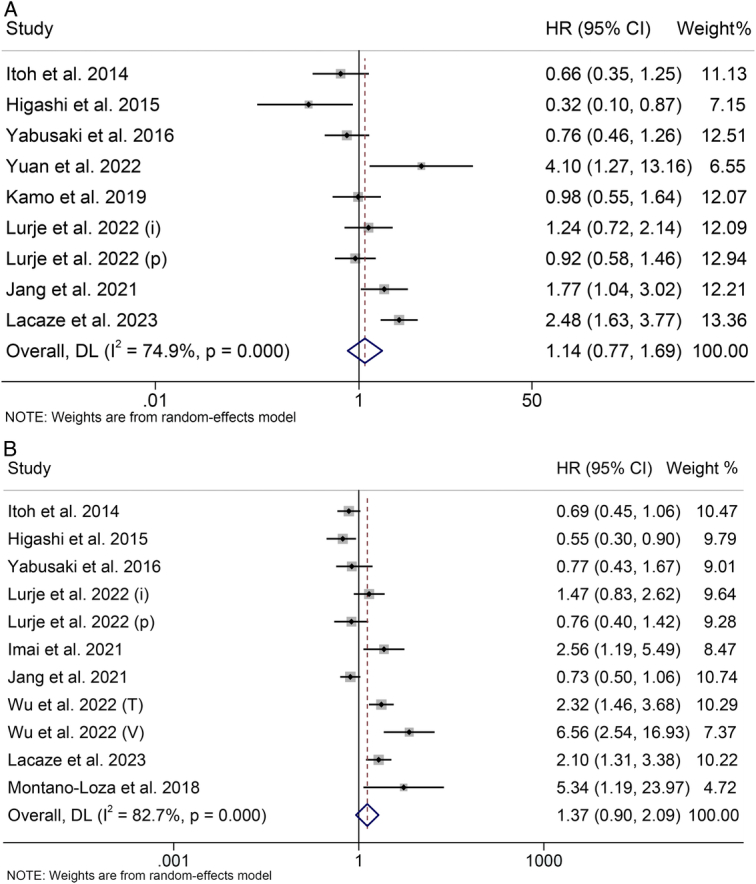
Forest plots of the relationship between visceral fat tissue and overall survival (A) and recurrence-free survival (B). CI, confidence interval; HR, hazard ratio.

The impact of pre-treatment visceral fat tissue on OS in patients undergoing hepatectomy or liver transplantation was investigated across nine studies, comprising 1492 participants. A random-effect model was employed, as indicated by the significant heterogeneity in the Cochran *Q* test and *I* ^2^ statistics (*I* ^2^=74.9%, *P*<0.001). The synthesized findings indicated there is no significant correlation between visceral fat tissue and OS (HR: 1.14, 95% CI: 0.77–1.69, *P*=0.501; Fig. 4A).

These findings were corroborated within these subgroups by either univariate or multivariate analyses and by either hepatectomy or liver transplantation (Table 3). Among the seven studies, visceral fat area (VFA) was employed to measure visceral fat tissue, while three studies utilized visceral fat index (VFI) for the same purpose. Notably, VFA exhibited no significant association with OS (HR: 0.92, 95% CI: 0.65–1.30, *P*=0.635; Table 3), whereas a high VFI demonstrated a significant correlation with shorter OS (HR: 2.18, 95% CI: 1.57–3.03, *P*<0.001; Table 3). The above findings also hold true in HCC patients, LD patients, ICC patients, and PCC patients (Table 3).

**Table 3:** Subgroup analysis of the association between visceral fat tissue and the outcomes after hepatectomy or liver transplantation.

		Test of association		Test of heterogeneity		
Variable	Included studies	HR (95% CI)	*P*	Modal	*I* ^2^	*P*
Visceral fat tissue (OS)						
Cox model						
Multivariate analysis	4	1.09 (0.49–2.39)	*P*=0.839	R	85.1%	*P*<0.001
Univariate analysis	5	1.07 (0.75–1.52)	*P*=0.722	R	46.3%	*P*=0.114
Treatments						
Hepatectomy	7	1.05 (0.68–1.64)	*P*=0.821	R	77.9%	*P*<0.001
Liver transplantation	2	1.82 (0.45–7.30)	*P*=0.399	R	78.8%	*P*=0.030
Visceral fat calculations						
Visceral fat area	7	0.92 (0.65–1.30)	*P*=0.635	R	52.9%	*P*=0.047
Visceral fat index	2	2.18 (1.57–3.03)	***P*** **<0.001**	R	0	*P*=0.330
Disease types						
HCC	4	0.80 (0.43–1.49)	*P*=0.485	R	72.8%	*P*=0.012
LD	2	1.82 (0.45–7.30)	*P*=0.399	R	78.8%	*P*=0.030
ICC	2	1.79 (0.91–3.53)	*P*=0.091	R	74.4%	*P*=0.048
PCC	1	0.92 (0.58–1.46)	*P*=0.723	–	–	–
Visceral fat tissue (RFS)						
Cox model						
Multivariate analysis	4	1.83 (0.81–4.13)	*P*=0.146	R	83.9%	*P*<0.001
Univariate analysis	7	1.20 (0.71–2.05)	*P*=0.496	R	84.1%	*P*<0.001
Treatments						
Hepatectomy	10	1.28 (0.84–1.96)	*P*=0.252	R	83.3%	*P*<0.001
Liver transplantation	1	5.34 (1.19–23.97)	***P*** **=0.029**	R	–	–
Visceral fat calculations						
Visceral fat area	5	0.79 (0.57–1.09)	*P*=0.149	R	38.9%	*P*=0.162
Visceral fat index	6	2.32 (1.22–4.42)	***P*** **=0.010**	R	84.8%	*P*<0.001
Disease types						
HCC	8	1.41 (0.81–2.46)	*P*=0.224	R	85.6%	*P*<0.001
ICC	2	1.82 (1.26–2.62)	***P*** **=0.001**	R	0	*P*=0.348
PCC	1	0.76 (0.40–1.43)	*P*=0.396	–	–	–

Bold values indicate statistically significant (P<0.05).

CL, confidence interval; HCC, hepatocellular carcinoma; HR, hazard ratio; ICC, intrahepatic cholangiocarcinoma; LD, liver disease; OS, overall survival; PCC, perihilar cholangiocarcinoma; R, random-effect model; RFS, recurrence-free survival.

### Preoperative visceral fat tissue and RFS

The relationship between visceral fat tissue and RFS was explored utilizing survival data from 11 studies involving 1807 participants. Figure 4B illustrates that there was significant heterogeneity observed among the studies (*I* ^2^=82.7%, *P*<0.001), warranting the application of a random-effect model. The findings demonstrated no significant correlation between visceral fat tissue and progression-free survival (PFS) (HR: 1.37, 95% CI: 0.90–2.09, *P*=0.141).

These results were validated through either univariate or multivariate analyses (Table 3). Similarly, VFA was not associated with PFS (HR: 0.79, 95% CI: 0.57–1.09, *P*=0.149; Table 3), while VFI was significantly associated with poorer PFS (HR: 2.32, 95% CI: 1.22–4.42, *P*<0.010; Table 3). The above findings also hold true in HCC patients and PCC patients (Table 3).

### Baseline visceral fat tissue and postoperative complications

**Figure 5:**
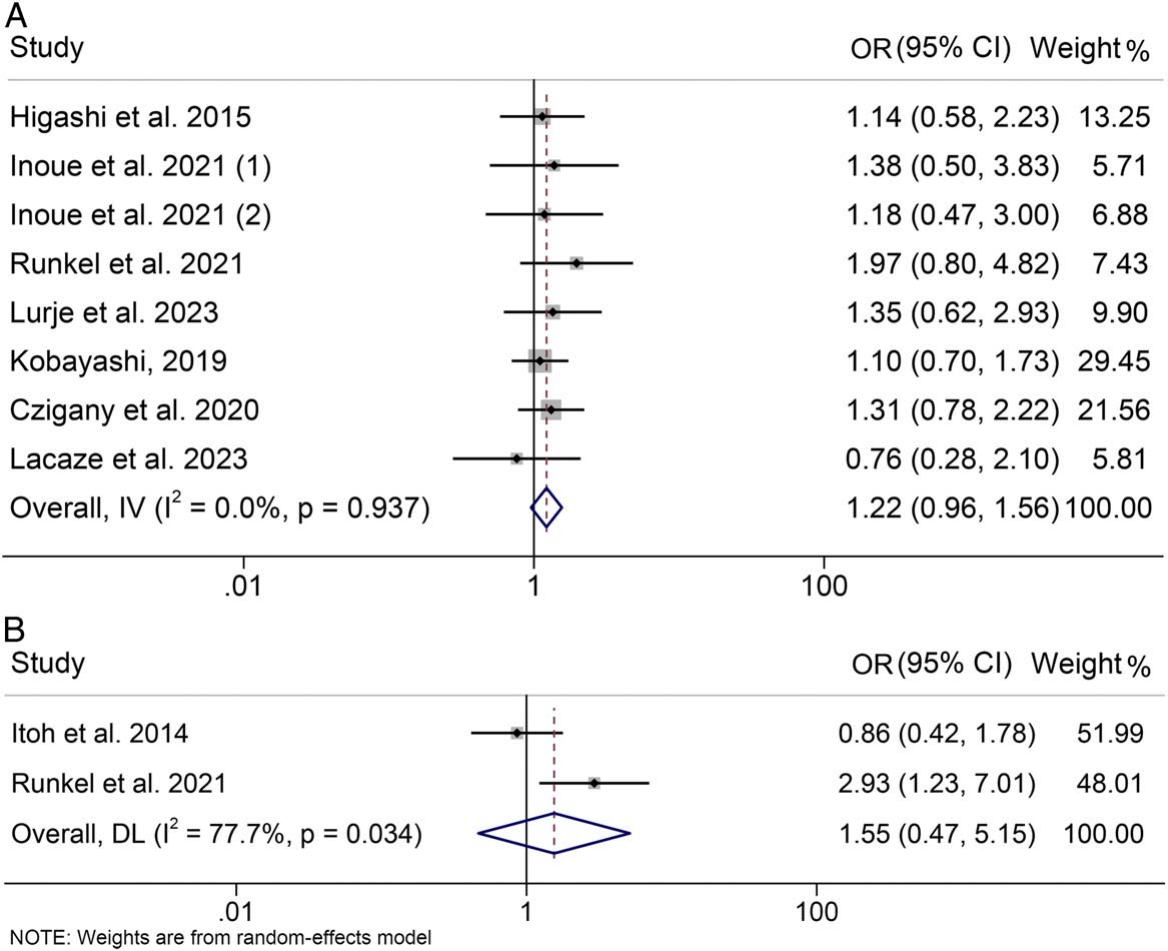
Forest plots of the relationship between visceral fat tissue and postoperative major complications (A) and postoperative complications (B). CI, confidence interval; OR, odds ratio.

Eight studies, encompassing 1577 participants, were incorporated into this analysis to investigate the influence of visceral fat tissue on preoperative major complications. Notably, no significant heterogeneity was observed among the included studies (*I* ^2^=0, *P*=0.937), prompting the utilization of a fixed-effects model. We found that high visceral fat tissue does not significantly increase the risk of major postoperative complications (HR: 1.22, 95% CI: 0.96–1.56, *P*=0.110; Fig. 5A). These findings were confirmed in subgroup based on both treatments and visceral fat calculations (Supplementary Table S1, Supplemental Digital Content 4, http://links.lww.com/JS9/C883). In addition, no correlation was found between visceral fat tissue levels and the risk of complications (HR: 1.55, 95% CI: 0.47–5.15, *P*=0.474; Fig. 5B). The above findings also hold true in HCC patients and PCC patients (Table 3).

### Correlation of subcutaneous fat index with overall and RFS

A total of three studies involving 383 patients were included in this analysis, examining the impact of preoperative subcutaneous fat index on OS or RFS. We found that patients with high subcutaneous fat index did not have significantly worse OS (HR: 0.65, 95% CI: 0.26–1.67, *P*=0.377; Supplementary Fig. S1A, Supplemental Digital Content 5, http://links.lww.com/JS9/C884) or RFS (HR: 0.97, 95% CI: 0.61–1.54, *P*=0.907; Supplementary Fig. S1B, Supplemental Digital Content 5, http://links.lww.com/JS9/C884) than patients with low subcutaneous fat index.

### Sensitivity analysis and publication bias

**Figure 6:**
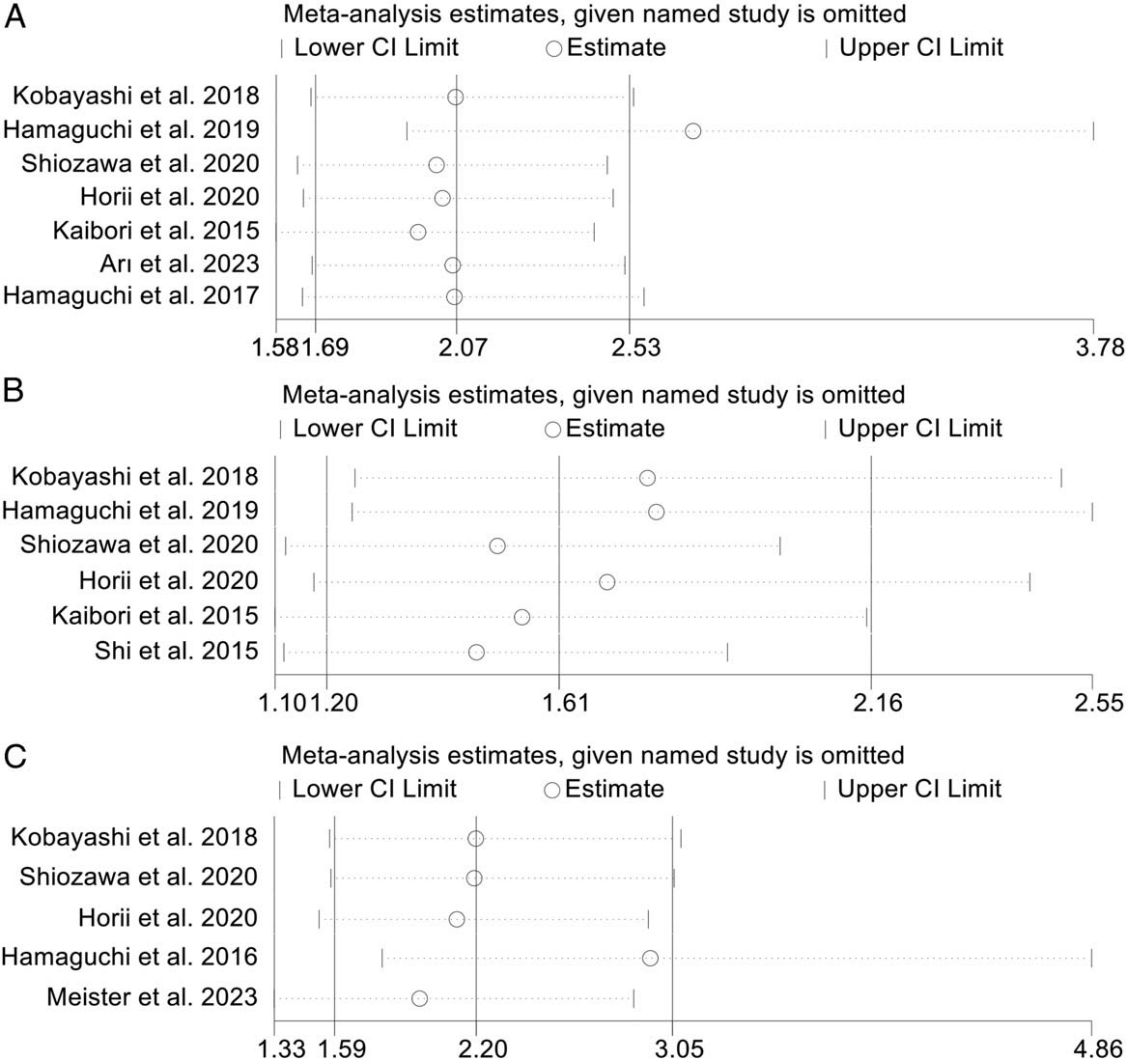
Sensitivity analysis of the association between intramuscular fat content and overall survival (A), recurrence-free survival (B), and postoperative major complications (C). CI, confidence interval.

Sensitivity analysis and investigations of publication bias were conducted initially to assess the robustness of the association between IMFC and survival outcomes and postoperative major complications. The exclusion of individual studies did not exert a significant impact on pooled HR for OS, which ranged from 1.96 (95% CI: 1.58–2.44, post-exclusion of Shi *et al*., 2015) to 2.71 (95% CI: 1.93–3.78, post-exclusion of Hamaguchi *et al*., 2019; Fig. 6A). Similarly, for RFS, the HR exhibited minimal variations, ranging from 1.46 (95% CI: 1.12–1.90, post-exclusion of Shi *et al*., 2023) to 1.78 (95% CI: 1.24–2.55, after excluding Hamaguchi *et al*., 2019; Fig. 6B). Besides, the pooled OR of the postoperative major complications remained unaltered upon the exclusion of any individual study (Fig. 6C).

The possibility of publication bias was assessed using Begg’s and Egger’s tests. Notably, the results of these tests did not indicate any significant publication bias for RFS (Egger’s test: *P*=0.106, Begg’s test: *P*=0.260) or postoperative major complications (Egger’s test: *P*=0.224, Begg’s test: *P*=0.221). However, Egger’s tests confirmed a significant publication bias in the case of IMFC and OS (Egger’s test: *P*=0.008, Begg’s test: *P*=0.133). To address this issue, we employed the trim and fill method, a valuable tool for estimating the number of potentially missing studies in OS. The results of this analysis indicated that even after incorporating these potentially missing studies, there was no discernible alteration in the pooled HR for OS (Fig. S2, Supplemental Digital Content 6, http://links.lww.com/JS9/C885).

**Figure 7:**
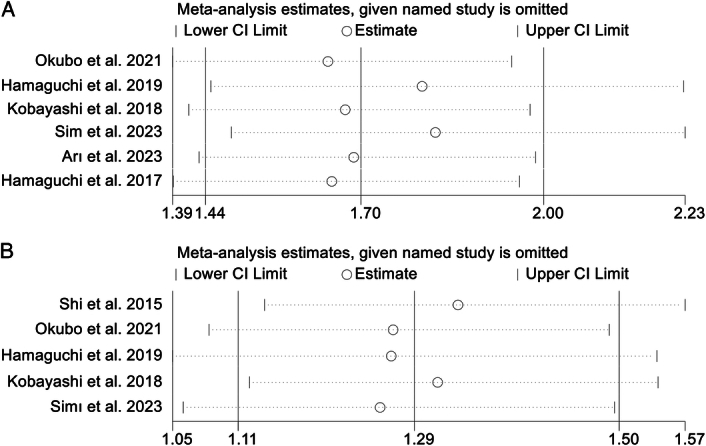
Sensitivity analysis of the association between visceral-to-subcutaneous fat tissue ratio and overall survival (A) and recurrence-free survival (B). CI, confidence interval.

In a similar vein, sensitivity analysis and assessment of publication bias have been employed to investigate the stability and reliability of the connection between VSR and survival outcomes. The HRs of the primary analysis remained unaltered upon the exclusion of any individual study (Fig. 7A, B). Rigorous scrutiny through Egger’s and Begg’s tests affirmed the absence of notable publication bias (OS, Egger’s test: *P*=0.106, Begg’s test: *P*=0.260; RFS, Egger’s test: *P*=0.346, Begg’s test: *P*=0.462).

Sensitivity analyses and assessments of publication bias were also conducted to evaluate the robustness of the association between visceral fat tissue and both survival outcomes and postoperative major complications. The exclusion of any individual study did not significantly influence the pooled results (Supplementary Fig. S3A–C, Supplemental Digital Content 7, http://links.lww.com/JS9/C886), and no evidence of publication bias was observed.

## Discussion

In this investigation, evidence from 31 studies has been compiled, demonstrating a substantial correlation between elevated VSR and IMFC with lower OS, reduced RFS, and an increased rate of complications in patients undergoing hepatectomy or liver transplantation.

Sarcopenia emerges as an adverse prognostic indicator across various cancer types^42,43^. While most prior investigations focused solely on skeletal muscle mass to assess sarcopenia, recent studies contend that this approach may not adequately capture lean muscle mass. The challenges lie in accurately quantifying adipose tissue within skeletal muscle using imaging techniques^44,45^. The European Working Group on Sarcopenia in Older People advocates a shift from defining sarcopenia based on muscle mass to prioritizing muscle strength or function^46^. Numerous studies underscore the significance of incorporating muscle strength testing or assessing IMFC^47^. Derived from skeletal muscle area, the psoas and skeletal muscle index may encompass both fat and muscle content, making it a potential limitation in capturing actual skeletal muscle mass. On the contrary, the determination of IMFC involves the computation of the skeletal muscle to subcutaneous adipose tissue ratio utilizing CT values. This method provides a more accurate representation of a patient’s sarcopenic condition.

Maintaining skeletal muscle homeostasis relies on the intricate equilibrium between protein synthesis and degradation^48^. Individuals with sarcopenia manifest heightened muscle catabolism^49,50^, likely attributed to chronic inflammation^51,52^. Currently, adipose tissue, along with skeletal muscle, is acknowledged as a secretory organ generating cytokines and adipokines. The interplay between myokines and adipokines assumes a pivotal role in diverse physiological pathways, including lipid metabolism, insulin resistance, and the attenuation of low-level chronic inflammation associated with obesity^53^. Our study reveals that high IMFC is strongly associated with a poorer prognosis and higher complication rates in patients undergoing liver surgery.

The functionalities of adipocytes exhibit distinctions between visceral adipose tissue and subcutaneous adipose tissue^54^. Visceral fat is acknowledged as an endocrine organ, releasing adipokines, leptin, and cytokines, including TNF-α and IL-6^55^. It harbors a greater quantity of cells, exhibits heightened vascularity compared to subcutaneous fat, and is characterized by an increased presence of inflammatory and immune cells^56^. Ong *et al*.^57^ discerned unique cell-surface markers of adipose-derived stem cells originating from visceral and subcutaneous adipose tissues. Many growth factors or cytokines, including insulin-like growth factor, vascular endothelial growth factor, hepatocyte growth factor, leptin, TGF-β, IL-8, and IL-10, are released by adipose-derived stem cells^58^. These factors have been implicated in the progression of cancer.

Tumor aggressiveness is increased by interactions between adipose-derived stem cells and cancer cells, as well as peritumoral adipocytes. Furthermore, these cells stimulate angiogenesis and peritumoral inflammation, which are crucial components of the tumor microenvironment^58^. Adipose tissue additionally exhibits immunological attributes, and in individuals with obesity, indications suggest a reduction in NK cells, substituted by inflammatory cells like macrophages^59^. Notably, visceral fat, unlike subcutaneous fat, directs proinflammatory cytokines into the liver and general circulation through drainage into the portal vein^60^. Our present study confirms that both visceral fat and subcutaneous adipose tissue are not associated with long-term patient prognosis or postoperative complication rates, whereas patients with high VSR have a higher rate of postoperative complications and a worse long-term prognosis.

The present analysis exhibits certain constraints. Firstly, the number of studies concerning subcutaneous fat index is limited, and there is an insufficient volume of research dedicated to exploring the impact of subcutaneous fat index on outcomes. Besides, the critical value of the same diagnostic indicator is not exactly the same in different studies. Hence, to attain more reliable conclusions, there is an urgent requirement for a worldwide, multicenter investigation to explore the impact of body fat tissue on outcomes in patients undergoing liver surgery.

## Conclusion

In summary, preoperatively high IMFC and VSR have the potential to forecast poorer OS and RFS and a higher risk of complications for patients undergoing hepatectomy or liver transplantation. Identifying patients who are at the highest risk before undergoing liver surgery might help surgeons optimize their perioperative strategy, which includes preoperative patient identification, surgical plan optimization, and prehabilitation. Implementing proactive measures can enhance the condition of patients by optimizing postoperative results. Integrating a systematic evaluation of IMFC and VSR using medical imaging before surgery can enhance the classification of liver surgical risks. This has the potential to reduce complications after surgery and provide enduring advantages for patients, such as improved survival rates.

## Ethical approval

Not applicable.

## Consent

Not applicable.

## Source of funding

This work was supported by grants from the National Natural Science Foundation of China (Nos 82172855 and 82370654) and the Natural Science Foundation of Hubei Province, China (2023AFB734).

## Conflicts of interest disclosure

The authors declared no conflicts of interest.

## Research registration unique identifying number (UIN)

1. Name of the registry: PROSPERO (International Prospective Register of Systematic Reviews).
2. Unique identifying number or registration ID: CRD42023447340.
3. Hyperlink to your specific registration (must be publicly accessible and will be checked): https://www.crd.york.ac.uk/prospero/#myprospero

## Guarantor

Weixing Wang.

## Data availability statement

The data that support the findings of this study are available from the corresponding author upon reasonable request.

## Provenance and peer review

We were not invited to this submission.

## Supplementary Material

**Figure:**
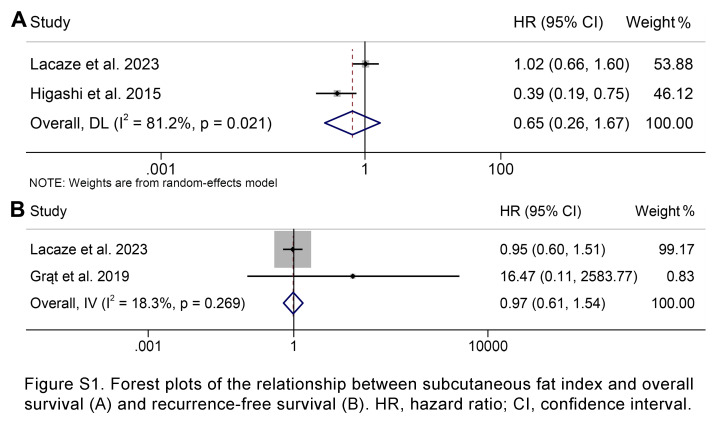


**Figure:**
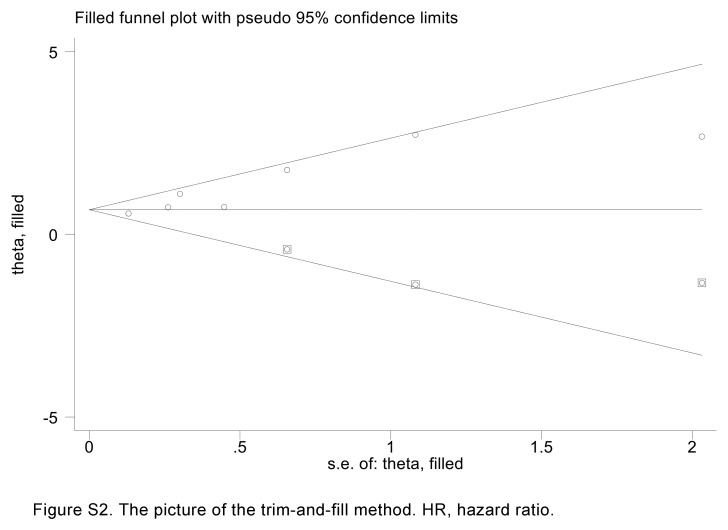


**Figure:**