# Supplementary material for: Influence of body fat tissue on outcomes in patients undergoing hepatectomy or liver transplantation: a systematic review and meta-analysis
Source: Int J Surg. 2024 Jun 26;111(1):1167–81. doi: 10.1097/JS9.0000000000001864 (PMC11745742; doi:10.1097/JS9.0000000000001864)
Supplement: Supplementary file 3 [file js9-111-1167-s003.docx]

Supplementary material 1. Search strategy

((Subcutaneous Adipose Index) OR (Subcutaneous Adipose Area) OR (Subcutaneous Adipose Tissue) OR (Subcutaneous Adipose Mass) OR (Subcutaneous Fat Index) OR (Subcutaneous Fat Area) OR (Subcutaneous Fat Tissue) OR (Subcutaneous Fat Mass) OR (Intramuscular Adipose content) OR (Intramuscular Adipose Index) OR (Intramuscular Adipose Area) OR (Intramuscular Adipose Tissue) OR (Intramuscular Adipose Mass) OR (Intramuscular Fat Index) OR(Intramuscular Fat Area) OR (Intramuscular Fat Tissue) OR (Intramuscular Fat Mass) OR (Intramuscular Fat Content) OR (Visceral Adiposity) OR (Visceral Adipose Index) OR (Visceral Adipose Area) OR (Visceral Adipose Tissue) OR (Visceral Adipose Mass) OR (Visceral Fat Index) OR (Visceral Fat Tissue) OR (Visceral Fat Mass) OR (Visceral Fat Area)) AND (((((((((((((Grafting, Liver) OR (Liver Grafting)) OR (Transplantation, Liver)) OR (Liver Transplantations)) OR (Liver Transplant)) OR (Liver Transplants)) OR (Transplant, Liver)) OR (Hepatic Transplantation)) OR (Hepatic Transplantations)) OR (Transplantation, Hepatic))) OR ("Liver Transplantation"[Mesh])) OR ((((((Hepatectomies) OR ("Hepatectomy"[Mesh])) OR (Liver Resection)) OR (Hepatic Resection)) OR (Liver Excision)) OR (Hepatic Excision) OR (Hepatectomy)))
