# Supplementary material for: Influence of body fat tissue on outcomes in patients undergoing hepatectomy or liver transplantation: a systematic review and meta-analysis
Source: Int J Surg. 2024 Jun 26;111(1):1167–81. doi: 10.1097/JS9.0000000000001864 (PMC11745742; doi:10.1097/JS9.0000000000001864)
Supplement: Supplementary file 4 [file js9-111-1167-s004.docx]

| **Table S1.** Subgroup analysis of the association between body fat tissue and complications after hepatectomy or liver transplantation | | | | | | | | |
| --- | --- | --- | --- | --- | --- | --- | --- | --- |
| Variable | Included studies | Test of association | | | | Test of heterogeneity | | |
|  |  | OR | 95%CI | *p*-value |  | Modal | I^2^ | *p*-value |
| **Intramuscular fat content (major complications)** | | | |  | |  |  |  |
| Treatments |  |  |  |  | |  |  |  |
| Hepatectomy | 4 | 1.96 | 1.33-2.88 | ***p* = 0.001** | | F | 0 | *p* = 0.426 |
| Liver transplantation | 1 | 2.93 | 1.60-5.36 | ***p* < 0.001** | | - | - | *-* |
| Disease type |  |  |  |  | |  |  |  |
| HCC | 1 | 1.77 | 1.15-2.72 | ***p =* 0.009** | | - | - | *-* |
| CRLM | 3 | 3.01 | 1.24-7.27 | ***p =* 0.014** | | F | 0 | *p* = 0.436 |
| LD | 1 | 2.93 | 1.60-5.36 | ***p* < 0.001** | | - | - | *-* |
| **Visceral fat tissue (major complications)** | | | |  | |  |  |  |
| Treatments |  |  |  |  | |  |  |  |
| Hepatectomy | 7 | 1.20 | 0.91-1.58 | *p* = 0.204 | | F | 0 | *p* = 0.893 |
| Liver transplantation | 1 | 1.32 | 0.78-2.23 | *p* = 0.308 | | - | - | *-* |
| Visceral fat calculations |  |  |  |  | |  |  |  |
| Visceral fat area | 7 | 1.26 | 0.98-1.62 | *p* = 0.075 | | F | 0 | *p* = 0.961 |
| Visceral fat index | 1 | 0.76 | 0.28-2.10 | *p* = 0.598 | | - | - | *-* |
| Disease type |  |  |  |  | |  |  |  |
| HCC | 2 | 1.09 | 0.81-1.46 | *p* = 0.571 | | F | 0 | *p* = 0.934 |
| CRLM | 3 | 1.36 | 0.90-2.05 | *p* = 0.147 | | F | 0 | *p* = 0.802 |
| ICC | 2 | 1.07 | 0.65-1.75 | *p* = 0.791 | | F | 0 | *p* = 0.380 |
| LD | 1 | 1.16 | 0.87-1.55 | *p* = 0.309 | | - | - | *-* |
| HCC, hepatocellular carcinoma; ICC, intrahepatic cholangiocarcinoma; PCC, perihilar cholangiocarcinoma; LD, liver disease; OR, odds ratio; CL, confidence interval; F, fixed-effect model. | | | | | | | | |
